# Comparison of *Palythoa tuberculosa*-dominated and hard coral-dominated benthic communities in Okinawa, Japan

**DOI:** 10.7717/peerj.21384

**Published:** 2026-06-18

**Authors:** Xavier Woodruff-Madeira, Mackenzie Stoeltje, David J. Die, James Davis Reimer

**Affiliations:** 1 Rosenstiel School of Marine, Atmospheric, and Earth Science, University of Miami, Miami, FL, United States of America; 2 Department of Marine Sciences, Chemistry and Biology, University of the Ryukyus, Nishihara, Okinawa, Japan; 3 Tropical Biosphere Research Center, University of the Ryukyus, Nishihara, Japan

**Keywords:** Coral reef, Zoantharia, Phase shift, Anthozoa, Community composition

## Abstract

Coral reefs worldwide are experiencing rapid declines due to the effects of climate change and other anthropogenic stressors. Losses of scleractinian hard corals in these ecosystems can lead to shifts in community compositions. Tracking these shifts is necessary to understand the current and future states of coral reefs undergoing such rapid change. In Okinawa, Japan, *Palythoa tuberculosa* is a common zoantharian species that can dominate the benthos in and around coral reefs. This study compared the compositions of hard coral-dominated benthic communities with those of nearby *P. tuberculosa*-dominated benthic communities at three Okinawa coral reef sites, representing among the first such datasets from the Indo-Pacific. Image data were collected from each site, and percent covers of different benthic categories were estimated based on stratified random point counts. PERMANOVA showed that benthic community composition differed significantly (*P* < 0.0001) between sites, and between coral reef and *P. tuberculosa* patch areas within sites. Specifically, hard coral-dominated communities supported more benthic diversity than the *P. tuberculosa*-dominated communities, with the absence of macroalgae in* P. tuberculosa*-dominated communities, and lower cover of hard and soft corals. Hard coral-communities hosted more benthic categories than *P. tuberculosa* communities across all sites. Further research pinpointing the causes of shifts in benthic dominance could help predict future outcomes of these ecosystems, contributing to coral reef conservation.

## Introduction

Global scleractinian hard coral coverage is declining on shallow coral reefs due to multiple anthropogenic factors, including climate change, overfishing, disease, and marine pollution (Aronson & Precht, 2001; Hoegh-Guldberg, 1999; Hughes et al., 2007; Pandolfi et al., 2003). Rising ocean temperatures have driven an increase in coral bleaching over the past several decades, threatening the long-term stability of these ecosystems (Pandolfi et al., 2011). Zooxanthellate scleractinian hard corals are foundational species for coral reefs, and their loss creates opportunities for other sessile benthic organisms to take advantage of coveted colonizable space, opening up the possibility for significant changes in benthic communities in the future (Fung, Seymour & Johnson, 2011; Hughes et al., 2003a; McCook, 1999; Norström et al., 2009; Stoddart, 1969). Such changes can result in phase shifts, in which a sudden change, or shift, occurs in the community composition of an ecosystem, and remains stable over time (Estes et al., 1998). The shift from hard coral-dominated coral reefs to algae-dominated ecosystems has been reported for some highly impacted reefs (Fung, Seymour & Johnson, 2011; Hughes et al., 2007; McCook, 1999). However, there are other possible benthic communities, such as those dominated by sponges, urchins, soft corals, corallimorpharians, actiniarians, or zoantharians (Kuguru et al., 2004; Norström et al., 2009; Bell et al., 2013; Reimer et al., 2021).

Zoantharia is an order of hexacorals found in a variety of marine environments, from coral reefs to deep water ecosystems (Burnett et al., 1997; Daly, Fautin & Cappola, 2003; Sinniger, Ocaña & Baco, 2013). Species from this order are often colonial, have soft bodies usually supplemented with sand and debris (Mueller & Haywick, 1995), and high growth rates (Burnett et al., 1997; Karlson, 1988; Silva et al., 2015; Shiroma & Reimer, 2010). The species *Palythoa tuberculosa* is abundant in Okinawa, and is the most common zoantharian around the island (Irei, Nozawa & Reimer, 2011), and is thought to be a generalist, able to rely more heavily on heterotrophy for energy, and flexibly host different Symbiodiniaceae types (Santos et al., 2021; Wee, Kobayashi & Reimer, 2021).

Some coral reefs in the Caribbean, and on the coasts of Brazil, Japan, and Hawaii, have experienced shifts to communities dominated by zoantharian species, including *Palythoa* species (Reimer et al., 2021), which form so-called “zoantharian barrens” (Reimer et al., 2021). On Okinawa, *P. tuberculosa* is known to form zoantharian barrens (Yang et al., 2013). Existing literature from Atlantic *Palythoa*-dominated communities suggests that they are less biodiverse and have a less complex physical structure than surrounding scleractinian-dominated coral reefs (Cruz et al., 2015a; Cruz et al., 2015b), but data from the Indo-Pacific are comparatively sparse.

To date, there is no consensus on the mechanism or mechanisms behind the shift from coral reefs to zoantharian barrens (Yang et al., 2013), although declines in water quality are often mentioned (Cooke & Mackie, 1976; Reimer et al., 2021). Detailed descriptions of ‘normal’ scleractinian coral communities and *P. tuberculosa*-dominated communities can provide an important foundation for a research dataset into these mechanisms. As there are few sites with data on the benthic community composition of *P. tuberculosa* patches, the purpose of this study was to describe these communities and compare them to nearby surrounding ‘normal’ coral reefs. The results will provide a starting point for future research on the dynamics between scleractinian and *P. tuberculosa* communities at these sites, as well as provide valuable datasets on *Palythoa* patches from the Indo-Pacific.

## Materials and Methods

### Data collection

**Figure 1:**
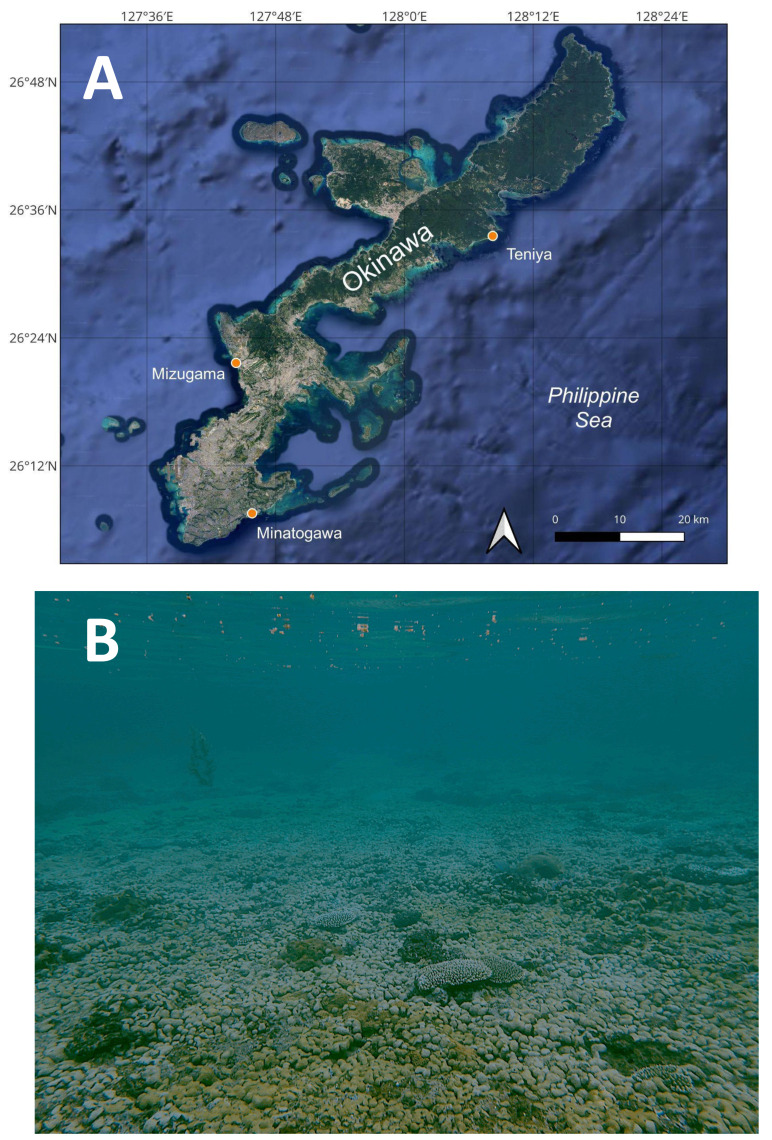
Map of research sites in this study and example of a Palythoa patch. (A) Location of the three study sites around Okinawa-jima Island, Japan examined in this study. Image generated from QGIS with an overlay from Google satellite images. (B) *Palythoa* patch at the Teniya site.

Surveys were conducted between June and August of 2024, just before the onset of a major bleaching event in Okinawa (Lalas et al., 2026). Three locations on the coast of Okinawa-jima Island were surveyed for this study; Teniya (26°33′46.8″N, 128°08′29.6″E), Mizugama (26°21′37.0″N, 127°44′20.1″E), and Minatogawa (26°07′15.3″N, 127°45′45.4″E) (Fig. 1A). An article by Yang et al. (2013) reported these three sites as supporting abundant *P. tuberculosa* populations. The surrounding coral reef communities were compared as benchmarks for ‘normal’ shallow water coral reefs in Okinawa. Most *P. tuberculosa* patches were at least approximately five meters in length along the greatest distance from one end of the patch to the other (Fig. 1B). All data collected from this study are available on coralnet.ucsd.edu/source/6128/ and as Supplementary Material for this article.

This study used a nested stratified random sampling design. Within each site two strata were defined, a scleractinian hard coral-dominated stratum and *P. tuberculosa*-dominated stratum. Within each stratum six random transects were surveyed, for a total of twelve transects per site. As *P. tuberculosa* is most common in shallow waters (<4.5 m; Irei, Nozawa & Reimer, 2011), surveys were conducted *via* snorkeling. Each survey sample was made of a 6 meter long transect within which six 0.5 m^2^ quadrats were evenly placed every 1 meter. Images were taken of each quadrat (=6 images per transect/patch; 36 images per site/strata, 72 images per site, 216 total images). Thus, in each transect a total benthic area of 3 m^2^ was surveyed. Transects were placed within scleractinian hard coral-dominated areas or across *P. tuberculosa-*dominated areas. Transects were placed in sites with minimal variation in depth, through the middle of either the coral-dominated area or *P. tuberculosa* area. Sample positions were selected haphazardly, by tossing the transect tape, without aiming, towards the benthos. After selecting sample positions, surveys were conducted in a single direction across the reef to reduce transit time during field work.

Data from the survey images were collected using a point count method in CoralNet (Williams et al., 2019; Beijbom et al., 2012; Beijbom, 2015; Beijbom et al., 2015; Chen et al., 2021; Courtney et al., 2022). As we noticed during initial analyses that approximately 10% of points were in shadow or unassignable to a category, and we aimed to have at least 30 valid points per image, 36 points were thereby randomly generated for each image, where each image represented one quadrat. Thus, our resolution of benthic cover estimates was 2.78%. Each point was assigned one of nine benthic cover categories following the methodology adopted by Yang et al. (2013); turf algae, crustose coralline algae, hard coral, *P. tuberculosa*, sand, rock and rubble, soft coral, macroalgae, other invertebrates, and sponges. Any points that fell on tape, quadrat, or shadow, or that fell outside the quadrat, were removed from subsequent analyses. For statistical analyses, a survey consisted of all the data points from the quadrat images of one transect. Data were recorded in MS Excel and then imported into RStudio for statistical analyses (R version 4.4.1, RStudio version 2024.9.0.375).

### Category proportions at each site

The mean percent coverages of each benthic category in each community type (hard coral and *P. tuberculosa*) were calculated at each of the three sites. These data were used to compare percentage coverage of benthic categories present in each community at each site. Comparisons were displayed with graphs created using the R package *ggplot2* (3.5.1).

### Nonmetric multidimensional scaling analysis

A nonmetric multidimensional scaling (NMDS) analysis was performed on the data to visualize similarities between benthic communities and between sites. Surveys were used as the sample unit for this model. The data were transformed into a distance matrix using the R package *vegan* (2.6-8) before running the NMDS model. The model was run in two dimensions (Oksanen et al., 2024).

### PERMANOVA

We used permutational multivariate analysis of variance (PERMANOVA) as variables involved did not fulfill parametric assumptions (Xu et al., 2022; Kramer et al., 2023; Murie et al., 2025). A PERMANOVA was run using the R Package vegan (2.6.8) to test for differences in the relative presence of benthic categories between community types and sites.

The variable community type (coral reef or *P. tuberculosa* patch) was nested within the variable site. Bray-Curtis dissimilarity was used to measure differences between sample variables. Statistical significance was assessed with 9,999 permutations.

## Results

### Category proportions at each site

**Figure 2:**
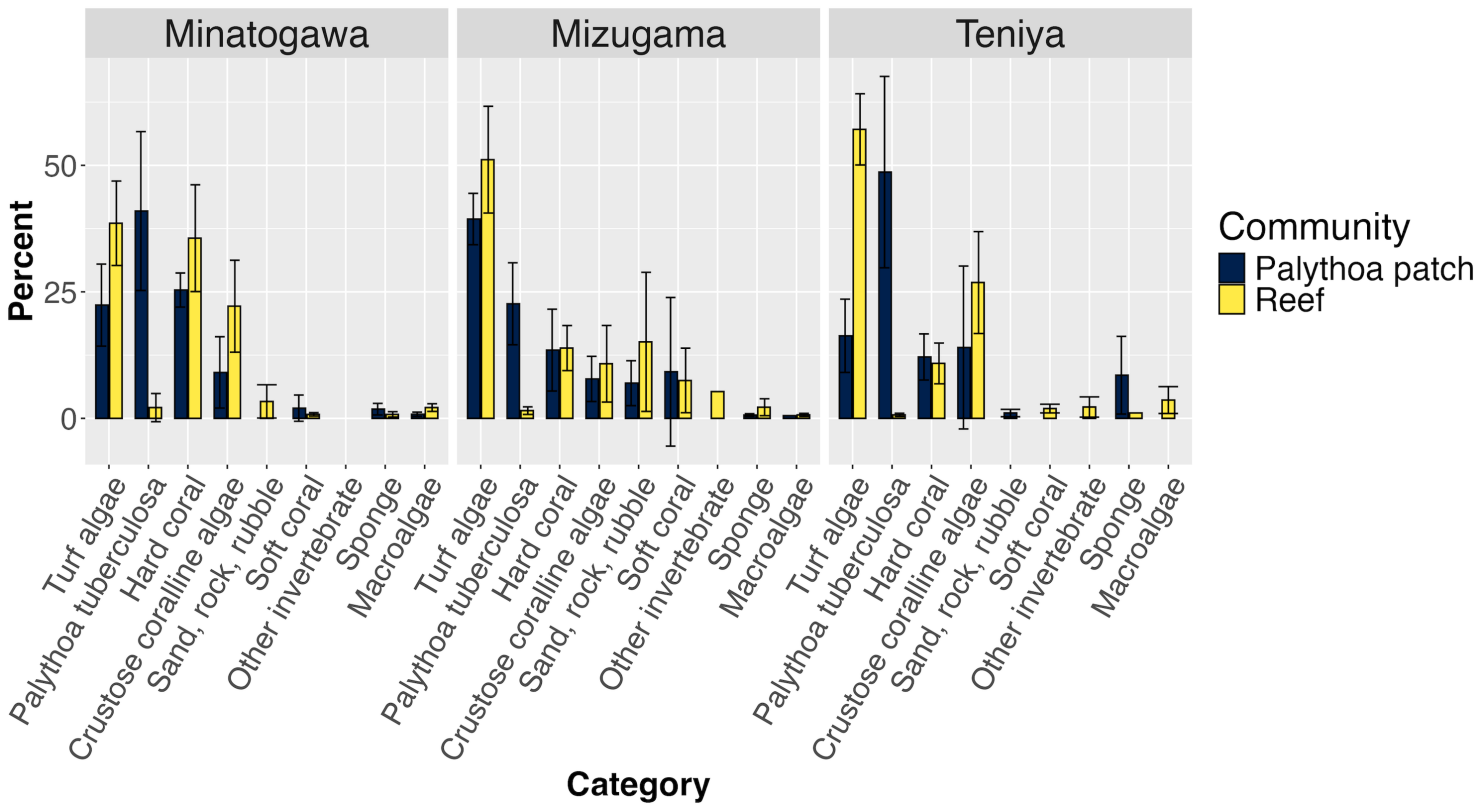
The means of the percentages of benthic cover of each benthic category in each community type for each site. Error bars represent the standard deviation.

**Figure 3:**
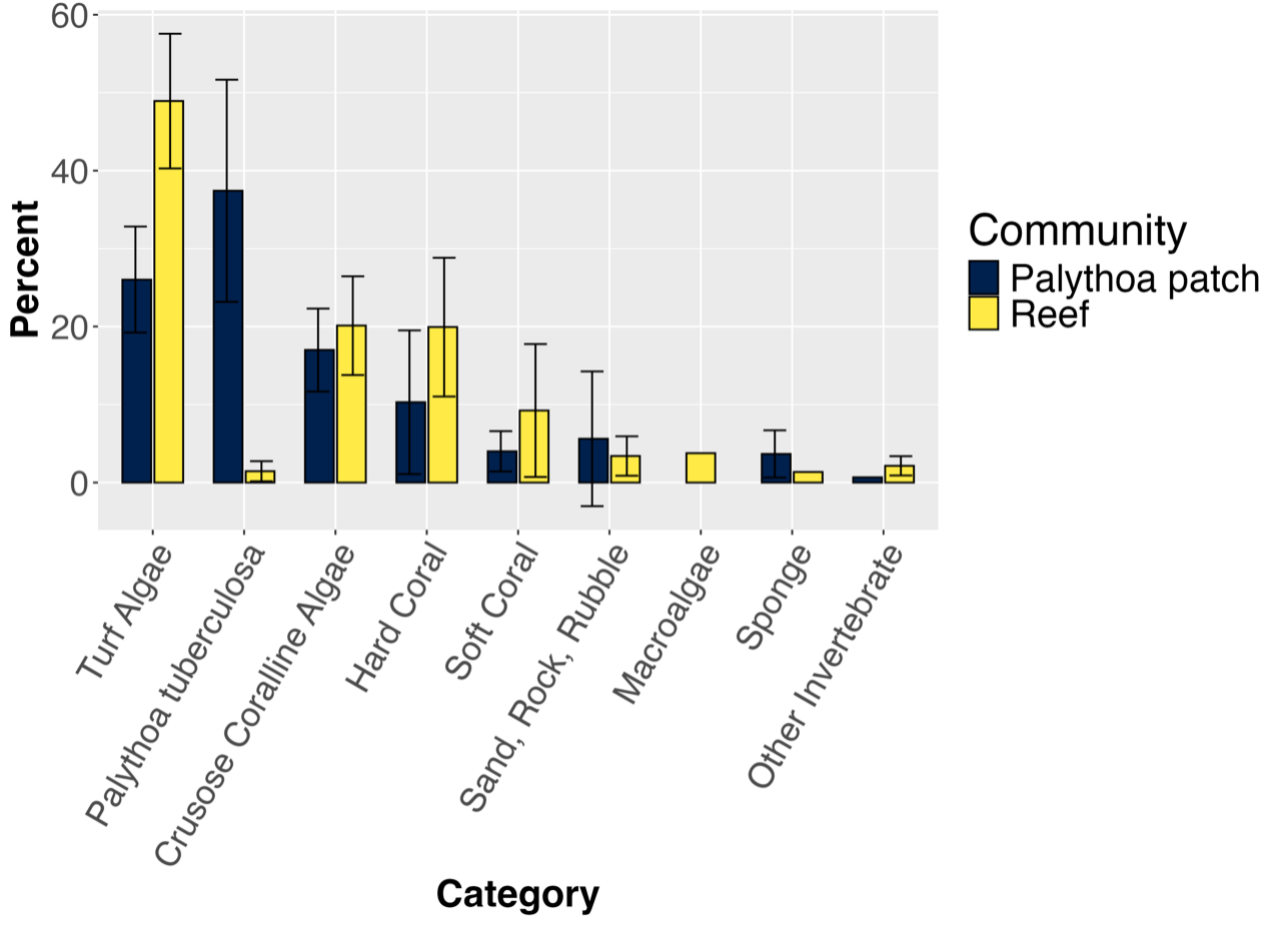
The means of the percentages of benthic cover of each benthic category in each community type. Error bars represent the standard deviation.

In total there were 6,900 points of data (216 images X 36 points per image, with tape, quadrat and shadows, *etc.* removed). Turf algae were the most abundant category at Mizugama (51.11% in hard coral community) and Teniya (57.12% in hard coral community), whereas in Minatogawa, *P. tuberculosa* was the most abundant category (40.98% in *Palythoa* patch), (Table 1, Fig. 2). Mizugama had the smallest percent coverage of *P. tuberculosa* within *P. tuberculosa* patch communities (22.64%) followed by Minatogawa (40.98%) and Teniya with the most (48.66%) (Table 1, Fig. 2). Other invertebrates, macroalgae, and sponge presence were negligible (<4%) except for sponges in *P*. *tuberculosa* community at Teniya (8.52%) and macroalgae in scleractinian coral reef community at Mizugama (5.29%) (Table 1, Fig. 2). The hard coral community hosted more benthic categories than the *P. tuberculosa* community across all sites (Figs. 2, 3). At Minatogawa the categories sand, and rock and rubble, were present in the hard coral but not the *P*. *tuberculosa* community, and at Mizugama the category “other invertebrates” was present in the hard coral and not the *Palythoa* community (Fig. 2). At Teniya soft corals, other invertebrates, and macroalgae were present in the hard coral and not in the *Palythoa* community, whereas sand, rock, and rubble was present in the *Palythoa* but not the hard coral community (Fig. 2). All three sites were dominated by turf algae, and turf algae cover in coral reefs was noticeably greater than cover in the *P. tuberculosa* benthos, especially at Teniya, where turf algae cover on the reef was 57.12% (Table 1, Figs. 2, 3).

**Table 1:** The means of the percentages of each benthic cover category in the *Palythoa tuberculosa* patch and coral reef communities at each of the three sites; Mizugama, Teniya, and Minatogawa.

**Site**	**Community type**	**Crustose coralline algae (%)**	**Hard coral (%)**	**Other invertebrates (%)**	***Palythoa tuberculosa*** ** (%)**	**Sponges (%)**	**Sand, rock, rubble (%)**	**Turf algae (%)**	**Soft coral (%)**	**Macro-algae (%)**
Minatogawa	*Palythoa* patch	25.36	9.08	0.82	40.98	1.82	2.02	22.39	0.00	0.00
Minatogawa	Reef	35.60	22.16	2.13	2.13	0.79	0.78	38.55	3.35	0.00
Mizugama	*Palythoa* patch	13.49	7.80	0.53	22.64	0.66	9.20	39.39	6.95	0.00
Mizugama	Reef	13.90	10.79	0.69	1.53	2.20	7.49	51.11	15.12	5.29
Teniya	*Palythoa* patch	12.14	14.01	0.00	48.66	8.52	0.00	16.33	1.06	0.00
Teniya	Reef	10.86	26.84	3.61	0.71	1.08	1.94	57.12	0.00	2.24

### Non-metric multidimensional scaling analysis

**Figure 4:**
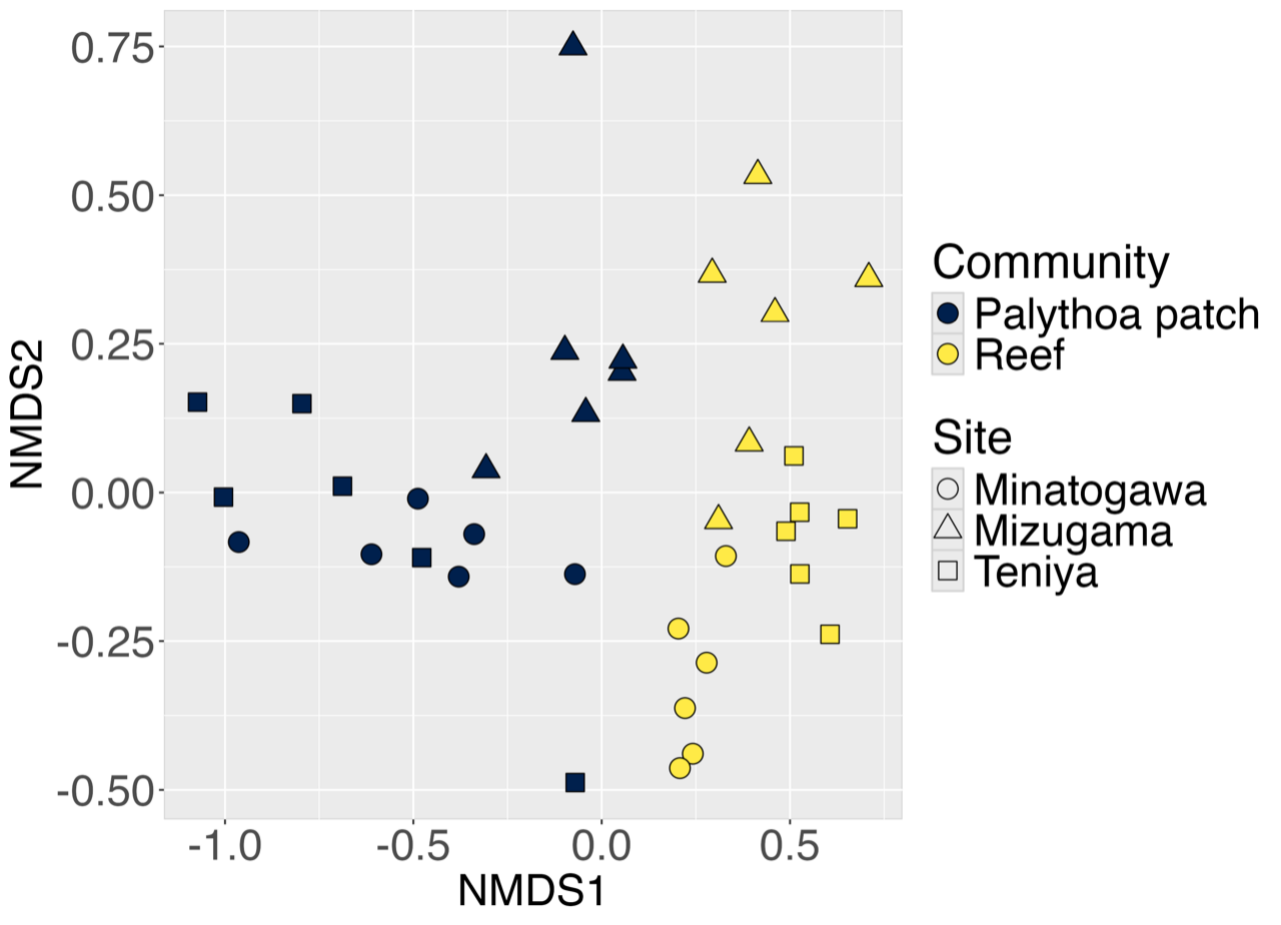
Ordination space of the NMDS model. Ordination space of the NMDS model where shape represents site and color represents community. The stress of the model was 0.122. Stress lies on a scale from 0 to 1, where stress levels <0.10 means the model represents the actual differences between sample units well.

The NMDS showed samples had a strong differentiation between the two benthic community types but did not have a similar magnitude of differentiation between the three different sites (Fig. 4). The distribution of the points based on community type showed a distinct pattern, where each type was segregated to one side of the graph, and there was no to little overlap (Fig. 4). There was no discernible pattern among the distribution of points based off of site on the graph (Fig. 4).

### PERMANOVA

There was a significant difference in composition of benthic categories between sites (*P* < 0.001), as well as a significant difference between community types (coral reef *vs P. tuberculosa* patch) within sites (*P* < 0.001) (Table 2).

## Discussion

The PERMANOVA results showed significant differences in coverage of benthic types between sites (*P* < 0.0001) and between community types (coral reef and *P*. *tuberculosa* patch) within sites (*P* < 0.0001). This difference was apparent visually in the NMDS, which showed a divide between the community makeup of hard coral and *P. tuberculosa* patches that we surveyed in this study. Hard coral communities supported one more benthic category in Minatogawa and Mizugama and three more categories in Teniya than the *P. tuberculosa* patch communities, indicating that these *Palythoa* communities may have lower biodiversity than nearby hard coral communities. Similar results have been reported from the Atlantic, where along the Brazilian coast Zoantharia-dominated sites showed lower benthic diversity compared to surrounding coral reefs (Soares et al., 2022).

Benthic cover of crustose coralline algae varied strongly among sites, suggesting that site-specific abiotic factors may influence benthic community composition in addition to differences between hard coral- and *P. tuberculosa*-dominated communities. Although Okinawa lacks many long-term fine-scale marine environmental datasets, recent research has demonstrated the importance of abiotic factors. For example, wave exposure has been shown to positively affect Sargassaceae macroalgae distribution (Abe et al., 2020), while salinity has been shown to potentially affect prokaryotic and eukaryotic eDNA communities at nearby Ishigaki Island (Hamamoto et al., 2025). Slightly lower salinity levels were also suggested to play a role in *Palythoa* barrens in a previous study at Mizugama (Yang et al., 2013), which we also examined in the current study. Other water quality variables may also be important; and while the mechanisms behind shifts from hard coral-dominated reefs to *P. tuberculosa* barrens has not yet been pinpointed, previous studies have indicated declines in water quality may be one factor (Yang et al., 2013; Reimer et al., 2021). It should be noted the *Palythoa* patches in this study were noted from surveys conducted in 2011 as mentioned in Yang et al. (2013), suggesting these patches represent ecological shifts that have been present for more than a decade and are not a transient state. Other research from Okinawa has shown distinct microbial communities from sandy bottoms, seagrass meadows, and coral reefs, suggesting biotic effects (Hamamoto et al., 2024). Similarly, our results indicated that some categories were more sensitive to the dominant anthozoan species than site location. Differences in the coverage of turf algae and hard coral between community types were greater than those between sites. Thus, the overall impacts of a shift from hard coral to *P*. *tuberculosa* barren are therefore likely to vary between sites. While site-specific abiotic factors could influence the outcome of a community shift, anthropogenic stressors such as ocean warming also may play a role in facilitating them (Hughes, 1994; Hughes et al., 2007). In the future, expanding similar research to coral reef and barren communities combining abiotic and biotic data into robust analyses could provide more insights into the roles both biotic and abiotic factors play in structuring communities.

**Table 2:** Results of the PERMANOVA test. Community type is nested within site.

**Treatment**	**Df**	**Sum of squares**	**R2**	**F**	**Pr(>F)**
Site	2	0.80	0.22	11.57	1e−04
Site:Community	3	1.82	0.50	17.52	1e−04
Residual	30	1.04	0.28		
Total	35	3.66	1.00		

Like many shallow coral reef scleractinian corals, *P. tuberculosa* hosts endosymbiotic Symbiodiniaceae, and are thus also susceptible to bleaching (Reimer, Takishita & Maruyama, 2006). Due to *P. tuberculosa*’s plasticity with symbionts, they may potentially survive heating events, and make a comparatively fast recovery (Noda et al., 2017; Wee, Kobayashi & Reimer, 2021). This could allow *P. tuberculosa* to colonize newly opened benthic space before new hard coral recruits have a chance to establish themselves in the following summer. Thus, *P. tuberculosa* barrens could be a transient state at longer time-scales, perhaps several years or even a decade or more, based on the results of Yang et al. (2013) and our results, showing barrens present at the Mizugama site in both 2011 and 2024, a span of 13 years. Typically the reefs around Okinawa island are kept cool during summer months by typhoons (Nadaoka et al., 2001). However, the island has seen several bleaching events within the last decade (Afzal et al., 2025), including no typhoons in 2024, and severe bleaching after the conclusion of our surveys (Lalas et al., 2026). If Okinawa continues to experience more regular bleaching events and an associated loss of hard coral cover, the number of *P. tuberculosa* barrens around Okinawa may increase, perhaps as well as increases incidences of other, non-accreting anthozoans (*e.g.*, Gösser et al., 2025).

The physical structures created by coral reef and *P. tuberculosa* patch benthic communities are distinct from one another. *P. tuberculosa* grows in wide and flat clumps, whereas hard corals and even many soft corals often have more verticality to their body plans and grow in a variety of shapes and structures. The structural complexity generated by coral reefs has been positively associated with biodiversity (Graham & Nash, 2013; Mazzuco, Stelzer & Bernardino, 2020). On the other hand, *P*. *tuberculosa* is comparatively much less rugose than most corals, with well-developed common tissue (coenenchyme) and is encrusting in form. Thus, the loss of this rugosity following a shift from hard corals to a *P. tuberculosa*-dominated community may then result in decreased biodiversity. *Palythoa* species have comparatively fast growth rates (Ronald., 1988; Silva et al., 2015), and harbor palytoxin, which could act as a deterrent against other sessile benthic species (Hadfield & Paul, 2001; Patocka et al., 2015; Simpson et al., 2004; Aratake et al., 2016). Thus, *Palythoa* could potentially reduce biodiversity *via* two methods; from a loss of species that typically live in spaces created by complex hard coral structures, and also from a loss of sessile benthic species that are outcompeted by *Palythoa* for benthic space. Additional impacts could include a reduction in coral rubble habitat production and reduced food sources for corallivores (Wolfe, Kenyon & Mumby, 2021).

The haphazard nature of the sampling in this study had the potential to introduce bias into the results, as the sampling locations were not determined using a strict randomization method. However, the survey area was comparatively large (108 m^2^), and the differences between true random sampling and haphazard sampling as in our study should not be impactful for limited observational studies such as this one. Additionally, the results of this study broadly agree with existing literature on Zoantharia barrens (Yang et al., 2013), showing reductions in coral cover (Yang et al., 2013; Cruz et al., 2015a) and of other biodiversity (*e.g.*, fishes; Cruz et al., 2015b). It is therefore likely that our results are broadly representative of the real shallow water hard coral and *P. tuberculosa* community compositions in Okinawa.

This study quantified the community composition of ‘normal’ coral-dominated areas and *P. tuberculosa* patches, and examined their differences at three sites around Okinawa Island. Our results provide a baseline description of two distinct community compositions at these sites. Shifts from scleractinian hard coral-dominated communities to *P. tuberculosa*-dominated communities could lead to decreases in biodiversity and species abundance. Testing these hypotheses is a logical next step. A more complete understanding of the causes of shifts from hard coral-dominated reefs to *Palythoa* barrens is needed, and longer-term monitoring of their impacts will better inform conservation efforts.

##  Supplemental Information

10.7717/peerj.21384/supp-1 Supplemental Information 1 Field data for comparison of *Palythoa tuberculosa*-dominated and hard coral-dominated benthic communities in Okinawa, Japan Includes site names, field survey dates, transect numbers, quadrat type, label codes, and annotator. Label codes as in text.

10.7717/peerj.21384/supp-2 Supplemental Information 2 R code for analyses of data for comparison of *Palythoa tuberculosa*-dominated and hard coral-dominated benthic communities in Okinawa, Japan

## Additional Information and Declarations

### Data Availability

The following information was supplied regarding data availability: The raw data and code are available in the Supplemental Files.
